# DeepSpaceDB: a spatial transcriptomics atlas for interactive in-depth analysis of tissues and tissue microenvironments

**DOI:** 10.1093/nar/gkaf1117

**Published:** 2025-10-29

**Authors:** Vladyslav Honcharuk, Afeefa Zainab, Yoshiya Horimoto, Keiko Takemoto, Diego Diez, Shinpei Kawaoka, Alexis Vandenbon

**Affiliations:** Laboratory of Tissue Homeostasis, Institute for Life and Medical Sciences, Kyoto University, Kyoto 606-8507, Japan; Department of Neuroscience, Graduate School of Medicine, Kyoto University, Kyoto 606-8507, Japan; Laboratory of Tissue Homeostasis, Institute for Life and Medical Sciences, Kyoto University, Kyoto 606-8507, Japan; Department of Breast Surgery and Oncology, Tokyo Medical University Hospital, Tokyo 160-0023, Japan; Department of Human Pathology, Juntendo University Faculty of Medicine, Tokyo 113-0033, Japan; Laboratory of Tissue Homeostasis, Institute for Life and Medical Sciences, Kyoto University, Kyoto 606-8507, Japan; Immunology Frontier Research Center, Osaka University, Osaka 565-0871, Japan; Department of Integrative Bioanalytics, Institute of Development, Aging and Cancer (IDAC), Tohoku University, Sendai 980-8575, Japan; Institute for Life and Medical Sciences, Kyoto University, Kyoto 606-8507, Japan; Laboratory of Tissue Homeostasis, Institute for Life and Medical Sciences, Kyoto University, Kyoto 606-8507, Japan; Institute for Liberal Arts and Sciences, Kyoto University, Kyoto 606-8507, Japan; Department of Mammalian Regulatory Network, Graduate School of Biostudies, Kyoto University, Kyoto 606-8507, Japan

## Abstract

Spatial transcriptomics enables detailed mapping of gene expression within tissues, revealing spatial organization of cellular and molecular processes. However, generating such data is costly and technically challenging, and analysis requires advanced bioinformatics skills. Although public datasets are growing, existing databases offer limited tools for interactive exploration and cross-sample comparison. Here, we introduce DeepSpaceDB (www.deepspacedb.com), a next-generation spatial transcriptomics database designed to address these challenges. The current version of DeepSpaceDB focuses on 10X Genomics Visium samples, ensuring higher-quality analyses and enhanced tools. This distinguishes it from databases that prioritize broad platform coverage over functionality. Emphasizing interactivity and advanced analytics, DeepSpaceDB enables flexible exploration of spatial transcriptomics data. Users can interactively compare gene expression across regions within or between tissue slices, such as between hippocampal areas of an Alzheimer’s model mouse and a control. The database also offers quality indicators, database-wide trends, and interactive visualizations like zoomable plots and hover-based info. Moreover, these functions are not restricted to samples in our database but can also be applied to samples uploaded by users. Combining advanced tools with interactive features, DeepSpaceDB is a powerful resource for spatial transcriptomics, enabling deeper insights into tissue organization and disease biology.

## Graphical Abstract

**Graphical Abstract:**
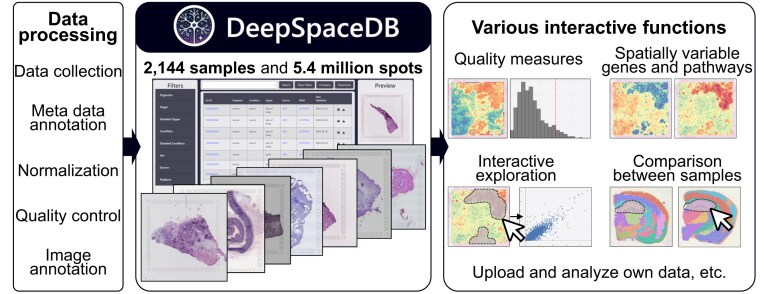

## Introduction

Spatial transcriptomics is a revolutionary technology that enables the spatial mapping of gene expression within tissues [1–3]. The retained positional information of cells is critical for understanding the complex organization of tissues and how cellular behaviors are influenced by their microenvironment. Thus, spatial transcriptomics provides unprecedented insights into the cellular and molecular architecture of biological systems, advancing our understanding of health, disease, and development.

However, generating new samples is financially costly and technically challenging. In addition, analyzing spatial transcriptomics data requires a high level of expertise in bioinformatics. At the same time, publicly available spatial transcriptomics samples are accumulating (see Supplementary Fig. S1A). There is therefore a demand for databases that allow users to easily and interactively explore existing spatial transcriptomics data. A number of spatial transcriptomics databases have been developed, including SpatialDB, SODB, STOmicsDB, and SOAR [4–7]. Although SpatialDB contains only a limited number of samples and appears to be no longer updated, SODB, SOAR, and STOmicsDB contain large numbers of samples covering many different platforms and therefore provide a great service as comprehensive data repositories. In addition, SORC and SCAR focus on spatial transcriptomics data across different cancer types [8, 9]. However, the use of these databases for exploring spatial transcriptomics data is limited because the tools they include are rudimentary and lack interactivity (see Supplementary Table S1). Whereas they allow users to plot the expression of a gene of interest or inspect clustering results, they lack—for example—measures of quality, the ability to freely compare gene expression between arbitrarily selected parts of tissue slices, or the ability for users to upload a sample and compare it to samples in the database. Standalone desktop tools such as Loupe Browser by 10X Genomics allow interactive analysis of single samples, including inspection of quality measures, gene expression, and clustering results. A few web platforms, including SRT-Server and spatialGE, have been developed [10, 11]. These platforms allow users to upload their own dataset of interest and perform various analyses without the need for programming expertise. However, both Loupe Browser and these web platforms lack the ability to browse through different samples, compare between manually selected regions, and inspecting pathway activities.

To address the above weaknesses, we present DeepSpaceDB, a spatial transcriptomics database that allows interactive and smooth exploration of spatial transcriptomics data. There are several critical points that set DeepSpaceDB apart from existing databases. First, compared to existing databases, DeepSpaceDB offers vastly expanded analysis functions with higher interactivity. DeepSpaceDB includes basic functions such as searching for a specific tissue and condition of interest and visualizing gene expression patterns in a tissue slice. In addition, DeepSpaceDB allows users to interactively select multiple parts within a tissue slice using their mouse cursor and compare gene expression between them. Comparisons can also be made between parts of different tissue slices, such as—for example—between the hippocampal parts of the brain of an Alzheimer’s disease model mouse and that of a healthy control mouse. All plots are interactive and allow users to zoom in and out and show additional information, such as gene expression levels or pathway activities, when hovering above spots with the cursor.

Second, DeepSpaceDB offers database-wide views and the ability to compare samples. This is in contrast with existing databases, which treat each sample essentially as an independent and isolated case. For example, quality indicators of samples can easily be compared to the database-wide trends, making it easier for users to judge whether a sample has sufficiently high quality. Furthermore, samples have been processed into 2D embeddings that facilitate the exploration of similar (i.e. nearby samples in the embedding) samples.

Third, DeepSpaceDB allows users to upload their own samples and apply many of the functions mentioned above to their own data, without the need for programming or data analysis experience.

The above advantages are possible only because we made the tradeoff to limit DeepSpaceDB to samples of a limited number of transcriptomics platforms; the current version includes only samples from the 10X Genomics Visium platform (currently the most widely used platform). In the future, samples from other popular platforms will be added. Note that this approach is the opposite of other spatial transcriptomics databases, which put more emphasis on wide coverage but with limited analysis and exploration functions.

With its unique combination of advanced functionality and user interactivity, DeepSpaceDB sets a new standard for spatial transcriptomics databases. As we expand to include more platforms, DeepSpaceDB is poised to become the go-to resource for the spatial biology community.

## Materials and methods

### Data collection and quality control

Transcriptome, image, and metadata were collected from the data sources, including NCBI GEO, the 10X Genomics website, and other data repositories (see Supplementary Table S2). Collected samples included both fresh frozen and formalin-fixed paraffin-embedded (FFPE) samples. Each sample was manually assigned a tissue of origin (using the Uberon anatomy ontology) [12] and condition information (Human Disease Ontology, DO) [13], as well as information related to the age or state of development, ethnicity or mouse strain, a link to scientific publications, and publication date, where possible. For each Visium sample, we inferred the Visium version (version 1: poly-A-based assays or version 2: probe-based assays) by comparing the genes present in the sample with the genes that are present in the probe sets as reported on the 10X Genomics website (https://www.10xgenomics.com/support/software/space-ranger/downloads). When the genes in a sample fit with the probe-based gene set the sample was labeled as version 2. When the genes in a sample contained genes that are not present in the probe-based assays (i.e. contained more genes) the sample was labeled as version 1.

Data from each sample was processed using the R Seurat package (version 5.0.1) [14]. Quality control included calculation of the number of detected genes and the UMI counts. After inspection (see the “Results” section), we employed a relatively loose quality filter, marking 56 558 spots (out of 5 417 362 spots, 1.04%) with no direct neighboring spots and spots with <100 detected genes as having low quality. Samples with >25% low-quality spots were filtered out. After removal of 30 low-quality samples, a total of 2144 samples remained (1361 human and 783 mouse).

### Transcriptome data processing

Each Visium sample was separately processed using a standard Seurat pipeline, including normalization, detection of highly variable genes, scaling, and Principal Components Analysis (PCA), followed by further dimensionality reduction using Seurat’s RunTSNE function based on the first 10 principal components (PCs), all using default parameters. Clustering of spots was done using the first 10 PCs using the FindNeighbors and FindClusters functions, using default parameters.

In each Visium sample, spatially variable genes (SVGs) were predicted using singleCellHaystack (version 1.0.2), SPARK-X (the SPARK package, version 1.1.1), and the binSpect method (the Giotto package, version 4.2.0) [15–19]. In brief, singleCellHaystack predicts features (genes) with nonrandom patterns of activity (gene expression levels) inside an input space. Here, we used the 2D spatial coordinates of the spots of each sample as input space to singleCellHaystack. To prioritize the detection of complex spatial patterns rather than gradual changes, the grid.points parameter of singleCellHaystack was set to a relatively high value (the number of spots divided by 7) but singleCellHaystack was otherwise used with default parameters. SPARK-X was run using the sparkx function using default parameters. The binSpect method was run after processing using functions normalizeGiotto and createSpatialNetwork, using the bin_method = “rank.”

The activities of biological processes were estimated using “module scores” as previously described [20]. In brief, we used the R package msigdbr (version 7.5.1) to define sets of genes associated with Gene Ontology (GO) biological processes [21]. For these gene sets, we calculated module scores using Seurat’s AddModuleScore function in each spot of all Visium samples, using default parameters. We predicted spatially variable pathways using singleCellHaystack, in the same way as we did for predicting SVGs, using the module scores of pathways as input.

For cell type deconvolution, we used robust cell type decomposition (RCTD; version 1.2.0) and SPOTlight (version 1.7.2) [22, 23]. We collected single-cell RNA-seq (scRNA-seq) reference datasets from CELLxGENE such that for each Visium sample we had a corresponding scRNA-seq dataset originating from the same (or a similar) tissue [24]. We limited reference datasets to data sequenced using 10X Genomics platforms to limit technical differences between Visium data and reference datasets. When multiple reference datasets were available, we gave preference to references that included common and abundant cell types of the tissue of interest, in addition to a variety of less common but biologically significant cell types. Many Visium samples are affected by medical conditions, such as cancer or immune diseases, and cell types and states in the reference datasets are likely to not match perfectly with the cell types and states in the tissue slices. Previous studies have shown how mismatches in cell types present in the reference dataset can reduce deconvolution performance [25, 26]. Improving the match between references and spatial transcriptomics datasets is one of our long-term goals. Here, for the current version of DeepSpaceDB, we limited ourselves to adding cancer cells to reference datasets. To do so, we first downloaded the tumor scRNA-seq data from the Tumor Immune Single-cell Hub 2 (TISCH2) database [27]. We then extracted malignant cells from these tumor tissue datasets and added these cells to the corresponding tissue scRNA-seq reference datasets, and used these augmented datasets as a reference for cell type deconvolution of Visium samples that were affected by cancer. Where needed, Ensembl IDs were converted to gene symbols using function mapIds of the R package AnnotationDbi (version 1.64.1) [28]. For RCTD, cell types for which there were <25 cells in a reference dataset were excluded, as RCTD requires at least 25 cells per cell type annotation. RCTD was run using doublet_mode=“full” and using otherwise default parameters. For SPOTlight, we used the workflow as described in the vignette for the Visium kidney sample here: https://marcelosua.github.io/SPOTlight/. In brief, in each reference dataset, marker genes for each cell type were calculated using the scoreMarkers function, models were trained using the trainNMF function, and applied to each Visium sample using the runDeconvolution function.

For spatial domain prediction, we used BASS (version 1.1.0.017) and BayesSpace (version 1.12.0), which have been reported to have relatively good performance on Visium data [29–31]. For BASS, we followed the workflow as described here: https://zhengli09.github.io/BASS-Analysis/DLPFC.html. To allow users the option to inspect a lower and higher number of domains, we ran BASS with the number of spatial domains set to 5 and 10. For BayesSpace, the function spatialPreprocess was run using the data as processed using Seurat, setting platform to “Visium” and otherwise default parameters. After that, function spatialCluster was run with the number of spatial domains set to 5 and 10.

For prediction of cell–cell communication, we used CellChat (version 2.2.0), which was reported to have an overall better performance than other tools [32, 33], following the workflow described here: https://github.com/jinworks/CellChat/blob/main/tutorial/CellChat_analysis_of_spatial_transcriptomics_data.Rmd.

### Database-wide transcriptome data processing

To allow for easier inter-sample and inter-location comparison, we integrated the collected data on two levels: the level of spots and the level of samples (pseudo-bulk). For the sample level, we calculated the average gene expression for each sample over all its spots, transforming it into a pseudo-bulk sample. We converted these pseudo-bulk samples into a Seurat object. This Seurat object was processed in a similar way to a typical single-cell dataset, including scaling, selecting highly variable genes (from the set of genes common to all samples), and dimensionality reduction using PCA and tSNE (using 10 PCs). Default parameters were used. We performed the spot-level integration in a similar way; for human and mouse samples separately, all spots of all samples were collected into a single Seurat object. After scaling and PCA, batch effects in this dataset were corrected using Harmony (version 1.2.0) using the first 50 PCs and treating each Visium sample as a batch [34], followed by dimensionality reduction using tSNE using the first 50 dimensions returned by Harmony, resulting in 2D tSNE embeddings of spots. To further annotate the variety of spots in the collection, we clustered spots into 50 clusters using k-means clustering and gave these clusters an annotation after inspection of the properties of spots they contain (tissue of origin, conditions, and gene expression). The overlap between clusters of spots and tissue and condition annotations was evaluated using the Jaccard index between each cluster and each annotation using the R package scmisc (version 0.8.4, https://github.com/ddiez/scmisc/). Jaccard index values were converted into *z*-scores using the means and standard deviations of Jaccard index values of 100 randomly permutated datasets.

### Image data processing and annotation

Using NDP.view 2.9.29 (Hamamatsu Photonics K.K.), a pathologist specializing in breast pathology annotated regions of invasive carcinoma, noninvasive carcinoma, normal ducts, vessels, tumor-infiltrating lymphocytes (TIL), and necrosis on the tissue sections. Areas where poor image quality made evaluation difficult were excluded from the annotations.

We used OpenAI’s GPT-4o for annotating the Visium tissue slice images as follows [35]. For all samples with “hires” Visium images, we first removed the 150 pixels at the top, bottom, right, and left sides, because the actual tissue slice is often located in the center of the image and not in its margins. The remaining cropped image was then cut into a 5-by-5 grid of rectangles of equal size. These 25 rectangles were passed on to GPT-4o using the Python openai package (version 1.36.0). We used the following prompt for samples with a known condition:

“This is a part of an H&E image of a <organism> tissue slice. The tissue is <tissue name>. The tissue is from a <condition> individual. Describe any tissue features or signs of pathology in at most 100 characters. If there is not enough tissue to say anything, write ‘empty’.”

where <organism> is “human” or “mouse,” <tissue name> is one of the tissue names as included in the Uberon ontology, and <condition> is one of the conditions included in the human DO. For samples lacking a condition annotation, the prompt was as follows:

“This is a part of an H&E image of a < organism > tissue slice. The tissue is <tissue name>. Describe any tissue features or signs of pathology in at most 100 characters. If there is not enough tissue to say anything, write ‘empty’.”

Responses were collected and are presented in the DeepSpaceDB database for each sample under section “Image annotation.”

### Data analysis using the tissue explorer tool

The Tissue Explorer tool allows users to compare gene expression and biological process activities between regions of interest within tissue slices. Regions of interest can be manually selected using the mouse cursor or can be based on spot clusters. The statistical significance of differences in expression or activities between regions of interest is evaluated using a two-sided *t*-test. *P*-values are adjusted for multiple testing using the Bonferroni correction.

### Searching the database using the search tool

The Search tool allows users to search the database using a gene or pathway of interest. Samples are returned sorted by the significance of their spatial pattern in each sample, as judged by the singleCellHaystack method (sorted by adjusted *P*-value). A one-sided Mann–Whitney U test is performed to evaluate the enrichment of samples originating from each tissue among top-ranking samples. Resulting *P*-values of the Mann–Whitney U tests are corrected for multiple testing using the Bonferroni method, and tissues are sorted by increasing adjusted *P*-value. The same test and ranking are conducted for conditions. Genes or pathways that have no discernible spatial pattern in any of the samples (singleCellHaystack adjusted *P*-value > 10^−10^ in all samples) are not included in this tool.

### Database construction

The backend implementation of DeepSpaceDB employs Flask, a lightweight WSGI web application framework written in Python. Flask was selected due to its ease of use, adaptability, and wide range of extensions. While keeping a simple core that allowed us to use it for our specific architecture, the framework offers necessary routing, request handling, and API development tools. Modern JavaScript is used in the frontend’s construction, utilizing current web development techniques. The main objective of the implementation is to create a dynamic and responsive user experience for our analysis tools while adhering to clean design standards.

The main relational database is SQLite, which was selected due to its transaction capability, dependability, and serverless design. Sample-specific spatial transcriptomics data is kept in CSV format, as it can be easily transferred and backed up, and it is directly compatible with analysis tools. Plotly, a complete graphing package that provides interactive scientific charts and graphs, support for Python and JavaScript, and a variety of plot types appropriate for visualizing spatial transcriptomics data, is used to construct the visualization layer [36].

## Results

### An overview of collected samples

**Figure 1.**
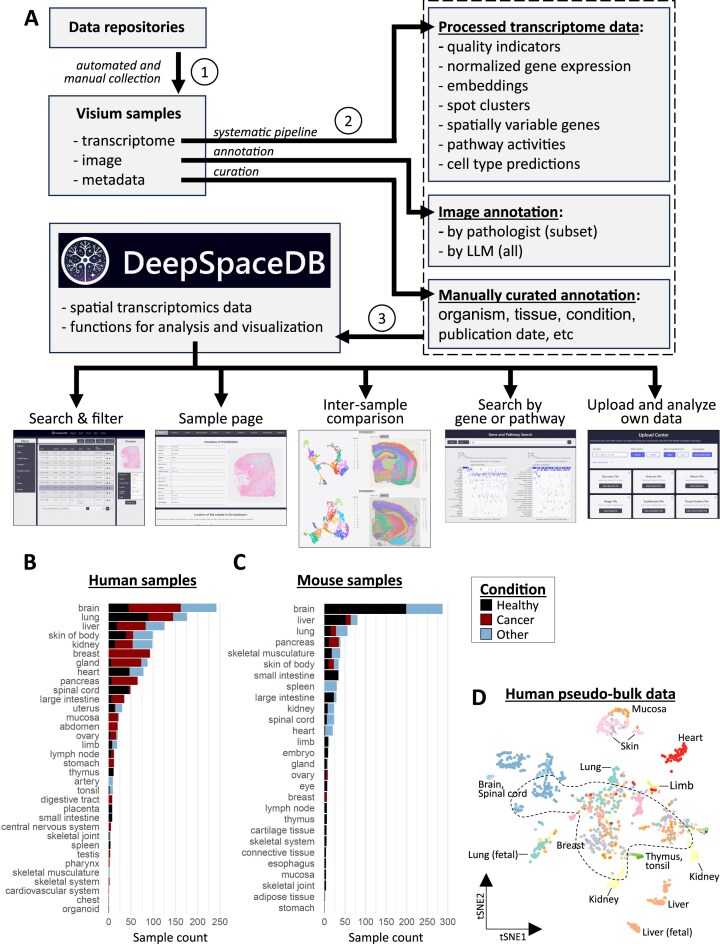
Spatial transcriptomics data collection of DeepSpaceDB. (**A**) Schematic overview of the data collection and processing workflow. First, transcriptomics, image, and annotation data were collected from various sources. Next, each data type was processed and analyzed. Finally, resulting data was included in DeepSpaceDB along with functions for analysis and visualization. (**B**) The number of human samples per tissue of origin. Colors indicate the condition. (**C**) Same as in panel (B) for mouse samples. (**D**) Embedding (tSNE plot) of human samples after processing to pseudobulk data. Colors indicate the tissue of origin. Prominent tissues are indicated. The dotted area indicates a large group of cancer-related samples (see also Supplementary Fig. S2B).

We collected Visium samples from NCBI GEO, the 10X Genomics website, and other data repositories (see Supplementary Table S2) and processed them through a pipeline that included initial processing, quality checks, normalization, and downstream analyses (Fig. 1A; see the “Materials and methods” section for details). Initial exploratory analysis of the collected data included a quality assessment. We observed a relationship between UMI counts and detected genes, similar to the tendency that is often observed for single-cell data (Supplementary Fig. S1B and C). However, it has been pointed out that these quality indicators at least partly reflect biological properties of the tissue of origin [37]. Here, too, we observed clear differences in the number of detected genes between different tissues (Supplementary Fig. S1D and E). In addition, we observed a strong association between these quality measures of each spot and its number of neighboring spots (Supplementary Fig. S1F–I). We labeled 56 558 spots (out of 5.4 million spots; 1.04%) with low numbers of detected genes and isolated spots (i.e. spots lacking direct neighboring spots) as having low quality. After filtering out 30 samples with >25% low-quality spots, a total of 2144 (1361 human and 783 mouse) Visium samples remained. Frequent tissues of origin included brain, lung, liver and skin for human, and brain and liver for mouse (Fig. 1B and C). Many human samples were related to diseases, especially cancer (Fig. 1B), and healthy human samples were rare. However, human “healthy” samples are likely to also include samples that simply lack a pathological annotation. Mouse samples contained more healthy control samples (Fig. 1C). In a second step of exploratory analysis, we processed each sample into a pseudo-bulk sample by averaging gene expression across all spatial locations and processed these into a 2D embedding (human: Fig. 1D, mouse: Supplementary Fig. S2A). We observed that, in general, samples from the same or related tissues tend to group together (i.e. have relatively similar gene expression patterns). However, especially for human samples, there is also a prominent group of samples originating from cancer patients (Fig. 1D, samples marked by dotted line; Supplementary Fig. S2B), where the grouping per tissue of origin is obfuscated.

We similarly explored the data on the level of individual spots. After clustering all human and mouse spots by similarity of gene expression into 50 clusters (see the “Materials and methods” section; Supplementary Fig. S2C and D), we attempted to assign a rough annotation to each cluster of spots based on their association with sample tissue and condition annotations, and the gene expression patterns within each cluster (Supplementary Fig. S3 for human and Supplementary Fig. S4 for mouse). Note that this annotation process is complicated by 1) the fact that spots reflect the average gene expression of multiple cells, resulting in few clearly distinct clusters, and 2) the lack of spot-level annotations (e.g. not all spots in a cancer-related sample are necessarily cancer tissue). Nevertheless, we could assign an annotation to most of the spot clusters and inspect the distribution of clusters with common and different annotations within the 2D embedding. For example, for humans, clusters associated with tumor tissue, tissue enriched in immune cells, neurons, and liver fill up distinct subspaces in the 2D embedding (Supplementary Fig. S5). Similar trends are seen for clusters associated with neurons, immune-enriched tissue, and liver in mouse (Supplementary Fig. S6).

Below follows a tour of the DeepSpaceDB database, including a case study exploring a human breast cancer sample, a comparison between pairs of samples, and searching the database with a query gene or pathway of interest.

### Case study: exploring a breast cancer sample

Under the Database tab, users can search the collection of samples for their species, tissue, condition, and other characteristics of interest. Selecting a sample shows a preview and takes the user to a sample-oriented page. Below, we will walk through this page and introduce its features. For this we will use a human breast cancer sample (DeepSpaceDB internal ID DSID000600), explore its features, and use it to compare between tumor and nontumor tissue regions in this sample [38].

#### Annotation data

This shows the background of the sample, including the data source (such as NCBI GEO, the 10X website, etc.), the tissue and condition (based on UBERON and DO or similar ontologies), the Visium version, and, where available, the sex, age, strain, or ethnicity of the patient or animal, and links to supporting scientific literature. A short description of the sample is also provided.

#### Location of the sample in the database

**Figure 2.**
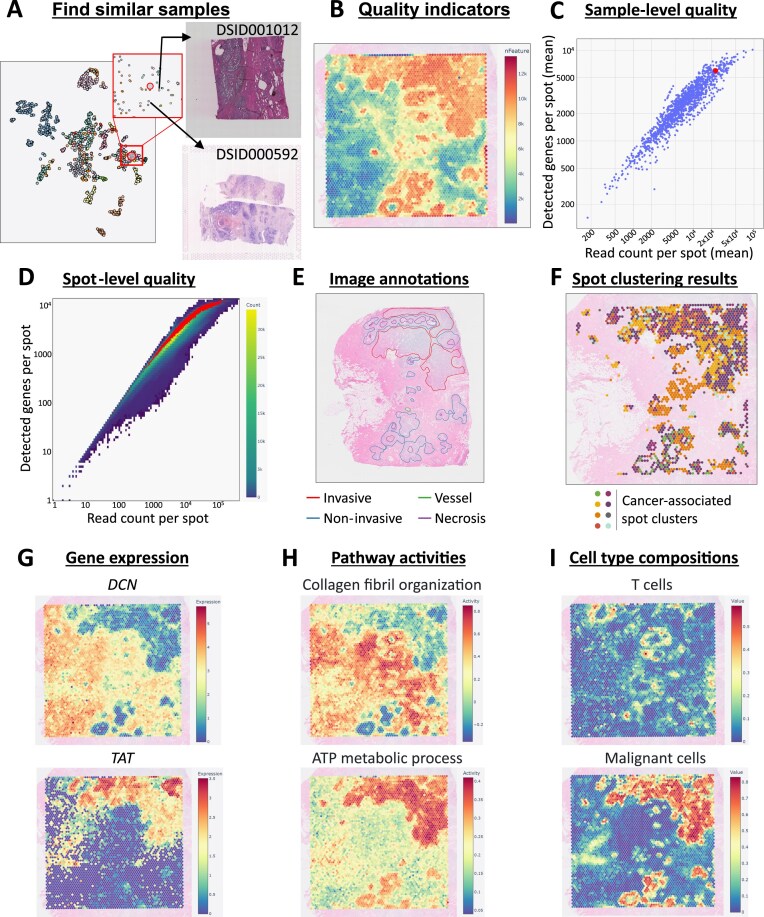
Inspection of a human breast cancer sample in DeepSpaceDB. (**A**) Using the 2D embedding (tSNE plot) to inspect samples that are similar to the currently selected sample in terms of gene expression levels. The images of two similar samples are shown as an example. (**B**) Plot of the number of detected genes at each location inside the tissue slice. (**C**) The average number of reads (*X*-axis) and the average number of detected genes (*Y*-axis) for all human samples in DeepSpaceDB. The currently selected sample is indicated in red. It has a relatively high number of reads and detected genes compared to other samples. (**D**) The number of reads (*X*-axis) and detected genes (*Y*-axis) per spot in the database. Spots of the currently selected sample are indicated in red. (**E**) The H&E image of the current sample with annotations by a human expert. (**F**) Spots in the current sample that were assigned to spot clusters associated with tumor tissue. Spots assigned to other clusters are not shown. (**G**) Examples of high-scoring SVGs: *DCN* and *TAT*. (**H**) Examples of high-scoring spatially variable biological processes. (**I**) Prediction of the distribution of T cells (top) and malignant cells (bottom) in the tissue slice.

One of the weak points of existing spatial omics databases is that samples are essentially treated and presented in isolation from each other, without integrating them into a database that allows inter-sample comparison. In DeepSpaceDB, however, all collected data has been integrated, allowing easier comparison between them. In this section, a 2D embedding is shown of all samples in the database, based on their gene expression profile after processing to pseudobulk data. The currently selected sample is indicated, allowing the user to quickly find samples with similar gene expression profiles. Here, for the currently selected breast cancer sample, we can see that similar samples include breast cancer samples, but also clear cell renal carcinoma, and ovarian and lung cancer samples (Fig. 2A).

#### Quality measures

It is often critical to confirm if a sample is of sufficiently high quality compared to other samples before using it to generate hypotheses. DeepSpaceDB offers various ways to check the quality of a sample and the spots it contains. The measures include the number of detected genes, the number of UMIs, the percentage of reads originating from mitochondrial genes, and the number of neighboring spots of each spot. These quality measures can be visualized within the tissue slice (Fig. 2B). In addition, the quality of a sample can easily be compared with all other samples in the database, a feature that is critically missing from existing databases. For example, we can see that the currently selected sample has a relatively high average number of reads per spot and average number of detected genes per spot compared to other samples in the database (Fig. 2C). The same comparison can be made on the level of individual spots (Fig. 2D).

#### Image annotations

Image annotations can assist the interpretation of spatial patterns inside tissues. In DeepSpaceDB (version 1.1), 69 human breast cancer samples have been manually annotated by a pathologist specializing in breast pathology. In the current sample, several invasive and noninvasive tumors were annotated, in addition to other features such as normal ducts and vessels (Fig. 2E). However, the manual annotation by a pathologist takes time and effort, and each pathologist tends to be specialized in one specific tissue. To collect manual annotations for all images in DeepSpaceDB is therefore a daunting task. Moreover, the Visium images are typically of lower resolution than typical images used for diagnosis. As an alternative, we therefore used an LLM to describe features inside each image, using a 5-by-5 grid. The LLM-based annotations of the current breast cancer sample are shown in Supplementary Fig. S7. Although the promise of AI-assisted annotation of histopathology images has been demonstrated [39], and these predictions can help interpret the spatial transcriptomics patterns, they need to be treated with care.

#### Spot clustering and spatial domains

Another way to assist the interpretation of spatial patterns of gene expression is to cluster spots by the similarity of gene expression patterns. In DeepSpaceDB, spots of each sample were clustered in two ways: (i) within-sample clustering and (ii) global clustering across the entire database (see the “Materials and methods” section and Supplementary Figs S3 and S4). Both clustering results can easily be visualized. We assigned annotations to the global clusters, which can assist in the exploration of structures and features within the tissue section. For example, for the selected breast cancer sample, we can see that spots assigned to tumor-associated clusters are accumulated at specific locations within the section (Fig. 2F). In addition, spatial domain prediction results by BASS and BayesSpace are available for every sample [29, 30]. Both methods predict spatial domains roughly reflecting the presence of tumor tissue within the tissue slice (Supplementary Fig. S8).

#### Spatially variable genes and biological processes

One advantage of spatial transcriptomics data is that it enables the visualization of gene expression within a tissue. In many cases, a subset of genes has a clearly distinct spatial expression pattern, reflecting substructures within the tissue slice. In DeepSpaceDB, SVGs in each sample have been precalculated by the singleCellHaystack, SPARK-X, and binSpect methods [15–17, 19]. Users can select their method of preference, search and select genes of interest, and instantly and interactively plot their expression patterns within the tissue slices along with the tissue image. In addition to individual genes, in DeepSpaceDB, the activity of sets of genes involved in a common biological process can be easily visualized (see the “Materials and methods” section). We used singleCellHaystack to predict the significance of the spatial pattern of activity of the biological processes in a similar way as we did for individual genes. As an example, in the selected breast cancer sample, top-scoring SVGs follow distinct patterns, exemplified by the genes decorin (*DCN*) and tyrosine aminotransferase (*TAT*) (Fig. 2G). Two of the high-scoring biological pathways in this sample are collagen fibril organization and ATP metabolic process, which have high activity in the non-cancerous and invasive tumor spots, respectively (Fig. 2H).

#### Cell type composition

For each sample in DeepSpaceDB, the cell type compositions at each spatial location were predicted using the methods RCTD and SPOTlight (see the “Materials and methods” section) [22, 23]. In brief, both methods use a reference scRNA-seq dataset to deconvolve the gene expression pattern of each Visium spot. Figure 2I shows how RCTD predicted the presence of T cells and malignant cells at specific locations within the tissue slice, fitting with the observations described earlier. Predicted cell–cell communication based on CellChat is also available [32].

### Interactive exploration of gene expression and biological process activity patterns

**Figure 3.**
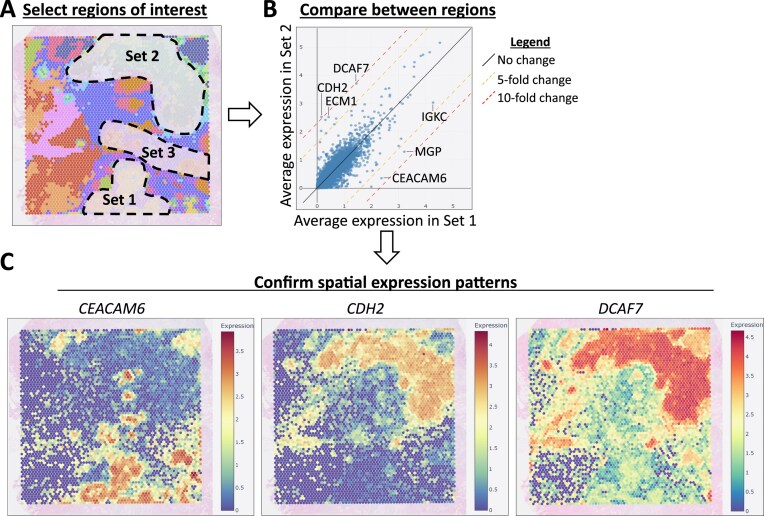
Comparing gene expression between different regions within a sample. (**A**) The three selected regions within the sample are indicated. Selections are made using the mouse cursor. (**B**) Scatterplot of the average expression of all genes in set 1 (*X-*axis) and set 2 (*Y-*axis). A number of genes with large differences are indicated. Similar comparisons between sets 1 and 3, and between sets 2 and 3 are shown in Supplementary Fig. S9. (**C**) The spatial expression patterns of three selected genes are shown. *CEACAM6* has higher expression in set 1 than in set 2. *CDH2* and *DCAF7* have higher expression in set 2 than in set 1.

The above exploration suggests that the currently selected sample contains an invasive tumor region at the top of the slice and a relatively smaller noninvasive tumor region at the bottom. We were interested in obtaining hints as to the difference between these two regions and the region located between them. DeepSpaceDB allows users to freely and interactively (using the mouse cursor) select multiple parts of a tissue slice and compare gene expression patterns between the selected parts. As an example, here we selected three parts within the tissue: the region at the bottom covering the smaller tumor region (“set 1,” 418 spots), the region at the top covering the larger tumor region (“set 2,” 1058 spots), and the region between the tumors (“set 3,” 461 spots) (Fig. 3A). DeepSpaceDB calculates the average gene expression in each of the selected parts and returns the results in the form of a table and scatter plot (Fig. 3B). *P*-values are estimated by a *t*-test based on the values of the spots in each selected region. Here, for example, we can see that *CEACAM6* has high expression in the smaller noninvasive tumor region at the bottom, while *CDH2* (N-cadherin) and *DCAF7* have high expression in the larger invasive tumor region at the top (Fig. 3C). While *CDH2* and *CEACAM6* are known to have elevated expression in various cancers [40–43], to the best of our knowledge no clear role or association between *DCAF7* and cancer has so far been described. This example illustrates the potential use of DeepSpaceDB for facilitating exploratory analysis and hypothesis generation. Similarly, we can easily find and inspect genes with large differences in expression between sets 1–3 (Supplementary Fig. S9A and B) and between sets 2–3 (Supplementary Fig. S9C and D). In addition to manually selected sets of spots, comparisons between spot clusters are also possible, as well as comparisons of biological process activities. For example, a comparison between spot clusters 2 and 9, which roughly overlap with the invasive and noninvasive tumor regions, respectively, identified several processes with differential levels of activities between these clusters (Supplementary Fig. S9E–G). The comparison results can not only be visualized as a scatterplot, but also as a volcano plot (Supplementary Fig. S9F). Processes related to glucagon metabolism (e.g. “response to glucagon”) and ATP synthesis are more active in the invasive tumor region, while processes related to anoikis (e.g. “regulation of anoikis”) are repressed in the invasive tumor region (Supplementary Fig. S9G). Other processes related to epithelial structure maintenance (e.g. “maintenance of gastrointestinal epithelium”) are more active in the noninvasive tumor region.

### Download data

At the bottom of the sample page, DeepSpaceDB allows users to download various data, including the image and transcriptomics data of the sample. We also provide data files of pre-calculated features, such as the predicted spatially variable genes and biological processes, cell type deconvolutions, spatial domain predictions, and Seurat objects. Functions for downloading data from multiple samples are also available on the “Database” tab.

### Comparison between two different tissue slices

**Figure 4.**
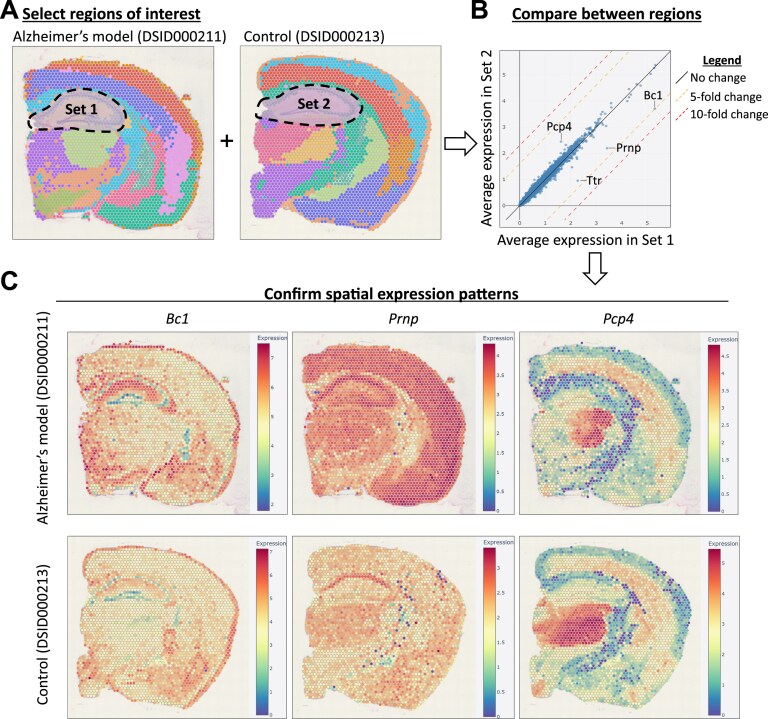
Comparing gene expression between two different samples. (**A**) The selected regions in an Alzheimer’s disease brain model (left) and a control brain (right) are shown. Note that spot colors reflect the clustering result of each separate sample and do not reflect matching tissue structures. (**B**) Scatterplot of the average expression of all genes in the sets of selected spots of the two samples. A number of genes with higher or lower expression in the Alzheimer’s disease model sample is indicated. (**C**) The spatial expression patterns of three selected genes are shown in the Alzheimer’s disease model samples (top) and in the control sample (bottom).

In addition to comparing gene expression between different parts of a single tissue slice, DeepSpaceDB also allows in-depth comparisons between two different tissue slices. Here we describe a comparison between the hippocampal part of an Alzheimer’s disease mouse model brain (sample ID DSID000211) and a healthy control brain (sample ID DSID000213), both from 7-month-old female mice [44]. Figure 4A summarizes the interactive selection using the cursor of the hippocampus of the two samples. The result of the comparison is a table with the average expression of all genes in both selections and a scatterplot summarizing the comparison (Fig. 4B). A number of genes have increased expression in the Alzheimer’s disease model, especially *Bc1, Prnp*, and *Ttr*, while *Pcp4* has a reduction in activity. *Prnp*, which encodes the cellular prion protein (PrPC), is linked to Alzheimer’s disease primarily through PrPC’s ability to bind toxic amyloid-beta oligomers and modulate synaptic dysfunction [45]. *Bc1* expression has been linked with impaired spatial learning and memory [46]. *Ttr* (transthyretin) has been shown to prevent amyloid formation and is thought to have a protective role [47, 48], but the increased expression of *Ttr* in this sample could not be confirmed in other Alzheimer’s disease model samples of the same study. *Pcp4* modulates Ca^2+^/calmodulin signaling but a direct role of *Pcp4* in Alzheimer’s disease has not been described. Inspection of the expression of these genes confirms the higher expression of *Bc1* and *Prnp* and the decreased expression of *Pcp4* in the hippocampus (and other regions) of the Alzheimer’s disease model brain (Fig. 4C).

### Searching DeepSpaceDB using a query gene or biological process

**Figure 5.**
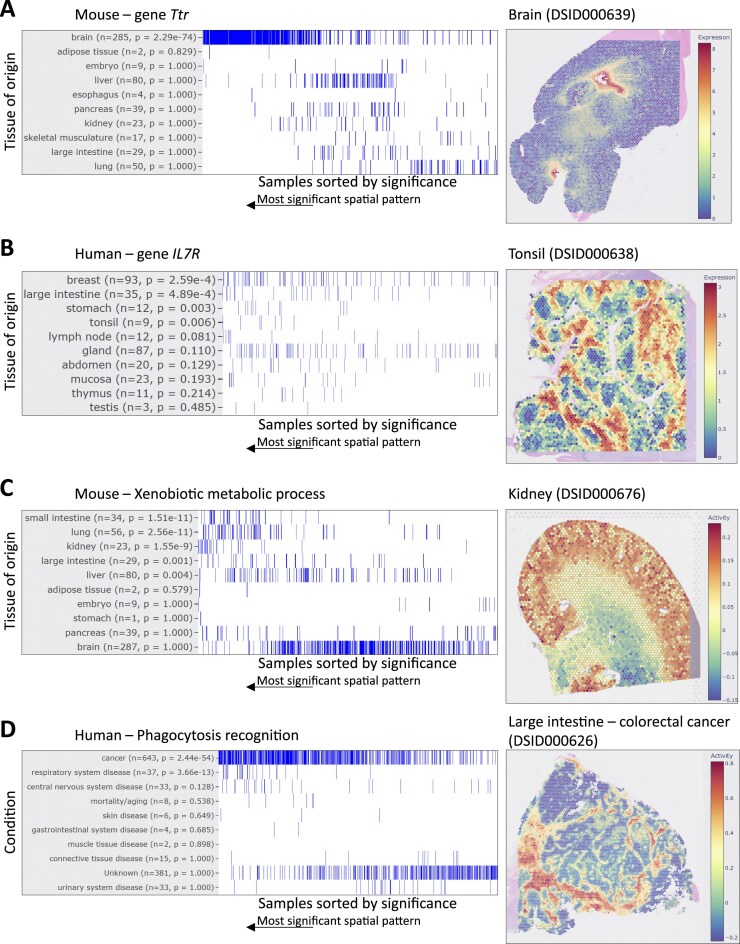
Searching DeepSpaceDB with a query gene or pathway. For each query a ranking of all samples sorted by significance of the spatial pattern of the query gene/pathway is shown on the left. Significant spatial patterns here refer to highly nonuniform spatial patterns as judged by the singleCellHaystack method. Tissues of origin or conditions are indicated, along with the number of samples (*n*) and the *P*-value of a one-sided Mann–Whitney U test (*p*), adjusted for multiple testing. Tissues or conditions are sorted by the *P*-value. Here, only the top 10 tissues or conditions are shown. On the right, one of the top 3 high-scoring samples (i.e. samples with strong spatial patterns for the gene or pathway in question) is shown with the expression/activity of the query gene/pathway. (**A**) Searching the mouse samples using query gene *Ttr*. High-scoring samples are strongly enriched in brain samples. (**B**) Searching the human samples using query gene *IL7R*. (**C**) Searching the mouse samples using query “Xenobiotic metabolic pathway.” High-scoring samples include small intestine, lung, and kidney. (**D**) Searching the mouse samples using query pathway phagocytosis recognition. High-scoring samples are biased toward cancer-related samples.

The above examples illustrate how to browse DeepSpaceDB starting from a tissue or condition of interest. An alternative is to search DeepSpaceDB using a query gene of interest, similar to the way biologists might search for a gene in literature or in a genome browser. As a result, DeepSpaceDB returns all samples in the database, ordered by the degree to which the query gene is an SVG as judged by the singleCellHaystack method. This allows users to find samples in which their gene of interest has varied expression within the tissue slice. Moreover, the ordering of samples and the tissue (or condition) they originated from can give valuable insights about the function of the query gene. The tool runs a one-sided Mann–Whitney U test on the ordering of samples of each tissue or condition and sorts them by the resulting *P*-value. Several examples are shown in Fig. 5 (left: summary of the sample ordering including the Mann–Whitney U test *P*-value for each tissue; right: a high-scoring sample). For the mouse *Ttr* gene, top-ranking samples are dominated by brain samples (Fig. 5A, left, *P*-value 2.29 × 10^−74^), such as sample DSID000639, where *Ttr* has highly concentrated expression in two locations within the brain (Fig. 5A, right). In contrast, for example, the human *IL7R* gene shows a complex expression pattern in a tonsil tissue slice (Fig. 5B, right), but there is only a weak tendency in the ranking of its tissues of origin (Fig. 5B, left).

In addition to searching using query genes, users can also use biological pathways as queries (Fig. 5C and D). For the xenobiotic metabolic process, high-scoring samples are enriched for small intestine (*P*-value 1.51 × 10^−11^), lung (*P*-value 2.56 × 10^−11^), and kidney (*P*-value 1.55 × 10^−9^). One of the top samples is a kidney sample where this pathway is more active in the renal cortex than in the renal medulla (Fig. 5C). This aligns with the known role of the proximal tubules inside the renal cortex in the biotransformation and active tubular secretion of xenobiotics and their metabolites. For the phagocytosis recognition biological process, there is no strong tendency among tissues of origin (not shown), but cancer-related samples tend to be among the high-scoring samples (Fig. 5D, left, *P*-value 2.44 × 10^−54^), possibly reflecting the activation of phagocytosis-related genes in these samples. One example is a colorectal cancer sample (Fig. 5D, right).

### Analysis of uploaded samples

Users can upload Visium data to DeepSpaceDB (see Supplementary Fig. S10 for a summary of the sample upload page). After uploading the necessary input files, data is processed in the same way as the public data included in our database, including normalization, calculation of quality indicators, dimensionality reduction, clustering, and prediction of SVGs. Moreover, users can compare the uploaded samples to samples in DeepSpaceDB. Uploaded data will be stored on the DeepSpaceDB server for a limited time, after which it is automatically removed. Users can also remove their data from the server at any time. Each uploaded sample can be accessed through a unique URL, which can be shared with collaborators, but data uploaded by users is not incorporated into the DeepSpaceDB data and is not made public to other users.

### Tutorial tab

The Tutorial tab contains short videos demonstrating how to use several functions of the database.

## Discussion

We present DeepSpaceDB, an integrated database for the interactive exploration and analysis of spatial transcriptomics data. Whereas existing databases focus on the collection of samples from many different platforms, DeepSpaceDB focuses on the implementation of tools that allow the user to explore spatial data interactively and intuitively, with the goal of gaining deep insight into the biological and clinical phenomena in the tissues and conditions included. To make this possible, we restricted the current version of DeepSpaceDB to data from the Visium platform, which has both a genome-wide coverage and many available samples. However, the field of spatial transcriptomics is still in rapid development, and it is reasonable to expect new platforms to be introduced in the next few years. We will monitor further developments and expand the database to cover future popular platforms, such as Xenium, Stereo-seq, or other platforms still under development. Undoubtedly, novel strategies will be needed to process the data of these higher-resolution platforms into formats that ensure a smooth experience for users and that allow harmonization across platforms.

DeepSpaceDB allows users to easily inspect the quality of samples (e.g. number of detected genes, read counts, etc.) and compare it with other samples, offering integrated views of the location of each sample relative to other samples, enabling users to easily find similar (or different) tissue slices. DeepSpaceDB also provides several levels of annotation: (i) spots have been grouped into database-wide clusters to which we have assigned annotations, (ii) an LLM was used to detect structural and pathological features in the image data of each sample, and (iii) high-quality annotations by a histology expert are available for a limited but increasing number of samples. Such annotations are absent in existing databases. Spatially variable genes have been pre-calculated in each sample, allowing for a smooth exploration by the user, without having to wait for each plot to be generated. Whereas the plotting of single genes is possible in existing databases, DeepSpaceDB also contains precalculated estimates of biological process activities in each sample. Here too, the statistical significance of spatial patterns of activity has been precalculated, allowing quick and easy visualization. Given the noisy nature of individual genes in spatial transcriptomics data, these pathway activities offer an additional tool for exploration to the users. Cell type predictions within each sample are based on curated reference single-cell datasets. Users are also able to interactively and freely select regions within tissue slices and compare gene expression patterns between these selected regions of interest. This can provide valuable hints about the difference in expression between—for example—tumors versus their immediate surroundings, between different parts of the brain, or between two different germinal centers inside a lymph node. Moreover, comparisons are also possible between different tissue slices. Users, therefore, can—for example—manually select the same part of the brain of a healthy control mouse and an Alzheimer’s disease model mouse and compare gene expression between these parts. Moreover, many of the above tools can also be applied to samples uploaded by the users. DeepSpaceDB can also be searched to find tissue sections that have a strong spatial pattern of expression for a given query gene of interest. The above functions allow users to interactively explore spatial transcriptomics data in a way that would be impossible to do in existing databases. Finally, raw and processed data as well as pre-calculated analysis results files are available for download for each sample, facilitating further downstream analysis by users.

DeepSpaceDB is primarily designed as an exploratory and hypothesis-generating resource. Its interactive tools allow researchers to rapidly survey thousands of spatial transcriptomics datasets, inspect quality metrics, and visualize spatial patterns without the need for local installation or computational expertise. While many users may still rely on offline frameworks such as Seurat for in-depth, publication-ready analyses of their own data, DeepSpaceDB provides a complementary environment for intuitive exploration and cross-sample comparisons. Importantly, all underlying raw and processed data, as well as pre-calculated results, can be downloaded directly from the database and used as input for offline workflows. In this way, the database serves both as a platform for discovery and as a starting point that can guide more detailed downstream analyses.

## Supplementary Material

gkaf1117_Supplemental_File

## Data Availability

Data of all samples can be downloaded from the DeepSpaceDB website. Links to the original data source are also provided for each sample.
