# Supplementary material for: DeepSpaceDB: a spatial transcriptomics atlas for interactive in-depth analysis of tissues and tissue microenvironments
Source: Nucleic Acids Res. 2025 Oct 29;54(D1):D1017–30. doi: 10.1093/nar/gkaf1117 (PMC12807613; doi:10.1093/nar/gkaf1117)
Supplement: gkaf1117_Supplemental_File [file gkaf1117_supplemental_file.pdf]

SUPPLEMENTARY DATA

DeepSpaceDB: a spatial transcriptomics atlas for interactive in-depth analysis of tissues and tissue microenvironments

SUPPLEMENTARY TABLES

Suppl. Table S1: Features included in DeepSpaceDB and other spatial transcriptomics databases and online platforms.

Feature	SORC	SCAR	SODB	SOAR	STOmicsDB	SpatialGE	SRT-server	DeepSpaceDB
Covers many platforms	yes	yes	yes	yes	yes	yes	yes	no
Includes quality indicators	no	no	no	no	yes	yes	yes	yes
Includes image data	no	no	no	yes	partly	yes	yes	yes
Includes image annotations	no	no	no	no	no	no	no	yes
Finding similar samples / overview of samples	no	no	no	no	no	no	no	yes
Gene expression visualization	yes	yes	yes	yes	yes	yes	yes	yes
Pathway activities	yes	yes	no	no	no	yes	yes	yes
Spot clustering	yes	yes	yes	yes	yes	yes	yes	yes
Cell type predictions	yes	no	no	yes	yes	yes	yes	yes
Cell-cell interactions	yes	no	no	no	yes	no	yes	yes
Interactive comparison of regions within a tissue slice	no	no	no	no	no	no	no	yes
Interactive comparison between slices	no	no	no	no	no	no	no	yes
Search for samples using a query gene	no	no	no	no	yes	no	no	yes
Upload and analysis of own samples	no	no	no	no	no	yes	yes	yes
Metadata filtering (organ, condition)	no	no	limited	yes	yes	NA	NA	yes
Download raw data	no	yes	no	limited	yes	NA	NA	yes
Disease coverage	cancer only	cancer only	various	various	various	NA	NA	various
Contains scRNA data	no	yes	no	no	yes	NA	NA	no
Database-wide gene /pathway search	yes	no	no	yes	yes	NA	NA	yes
Requires registration	no	no	no	no	no	yes	yes	no
No. of samples	269	41	3,145	2,785	15,601	uploaded sample only	uploaded sample only	2,144

Suppl. Table S2: Sources of spatial transcriptomics samples used for DeepSpaceDB.

Source name	URL	Number of samples
NCBI Gene Expression Omnibus	www.ncbi.nlm.nih.gov/geo/	1664
EMBL-EBI	www.ebi.ac.uk/biostudies/	205
Zenodo	zenodo.org	131
10X Genomics	www.10xgenomics.com/datasets/	58
Heart Cell Atlas	www.heartcellatlas.org/	42
Lung Cell Atlas	www.lungcellatlas.org/	12
Mendeley Data	data.mendeley.com	8
Reproductive Cell Atlas	www.reproductivecellatlas.org/	8
Welcome Sanger Institute	treg-gut-niches.cellgeni.sanger.ac.uk	8
internal	-	8

SUPPLEMENTARY FIGURES

(next page) **Suppl. Figure S1:** Tendencies of the samples included in DeepSpaceDB version 1.1. **(A)** The cumulative number of Visium samples included in DeepSpaceDB version 1.1 in function of their publication data. **(B-C)** Distribution of the number of reads per spot (X axis) versus the number of detected genes per spot (Y axis) for human (B) and mouse (C) samples. Colors indicate the number of spots per bin. **(D-E)** Boxplots of the number of detected genes per spot (X axis) for each tissue of origin (Y axis) for human (D) and mouse (E) samples. There are considerable differences between the tissues, although the tendencies have to be interpreted carefully because differences can also be to some degree caused by differences in sequencing depts between studies. **(F-G)** Boxplots showing the number of detected genes per spot (Y axis) in function of the number of immediate neighboring spots a spot has (X axis), for human (F) and mouse (G) samples. The number of immediate neighboring spots ranges from 0 (spots with no neighboring spots; i.e., isolated spots) up to 6 (spots with the maximum number of neighbors in the hexagonal grid of the Visium platform). **(H-I)** The same as (F-G) but showing the distribution of the number of reads per spot in the Y axis, for human (H) and mouse (I) samples.

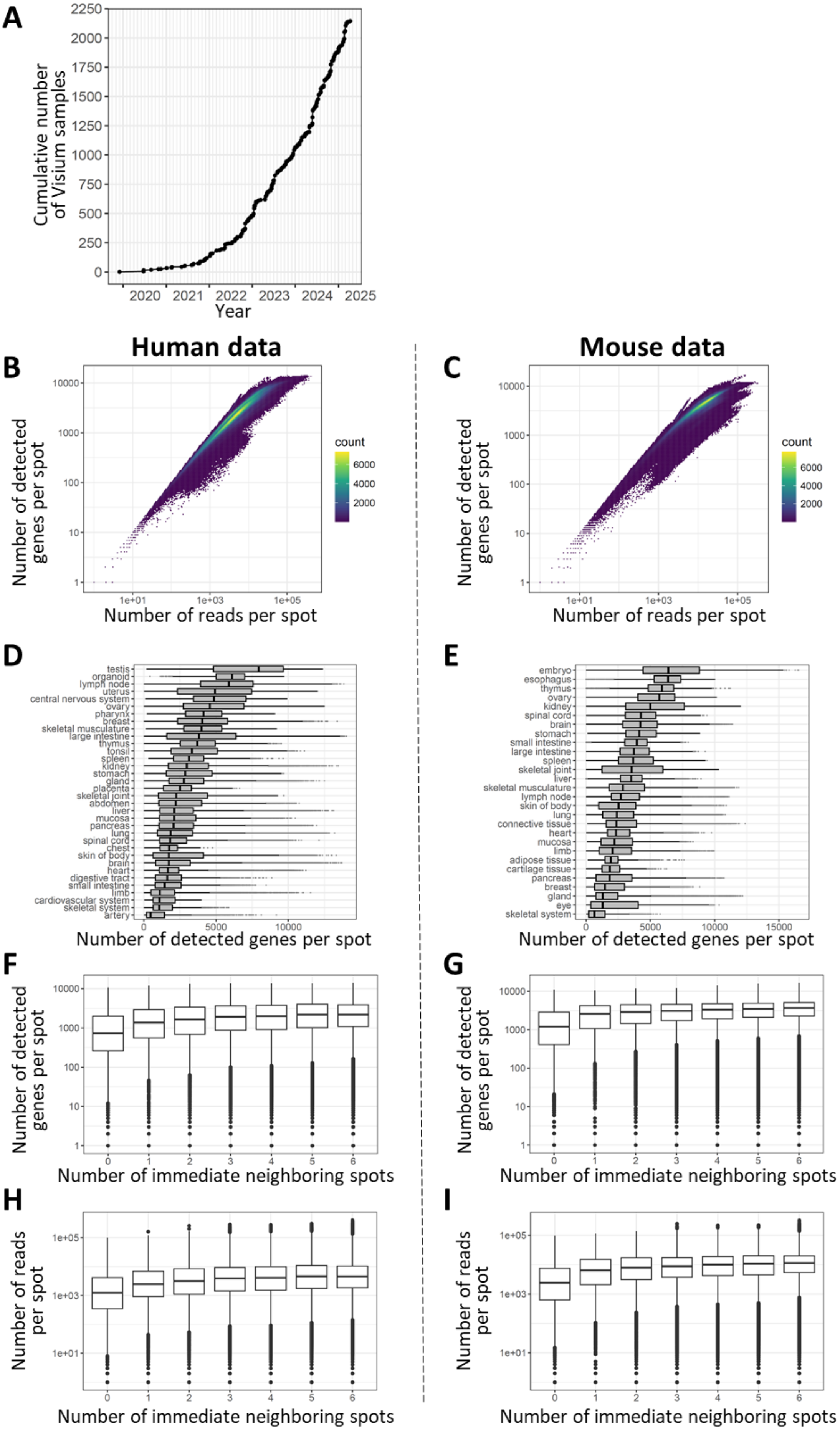
A
Cumulative number of Visium samples
Year
Human data
Mouse data
B
C
Number of detected genes per spot
Number of reads per spot
count
D
E
Number of detected genes per spot
F
G
Number of immediate neighboring spots
H
I
Number of reads per spot

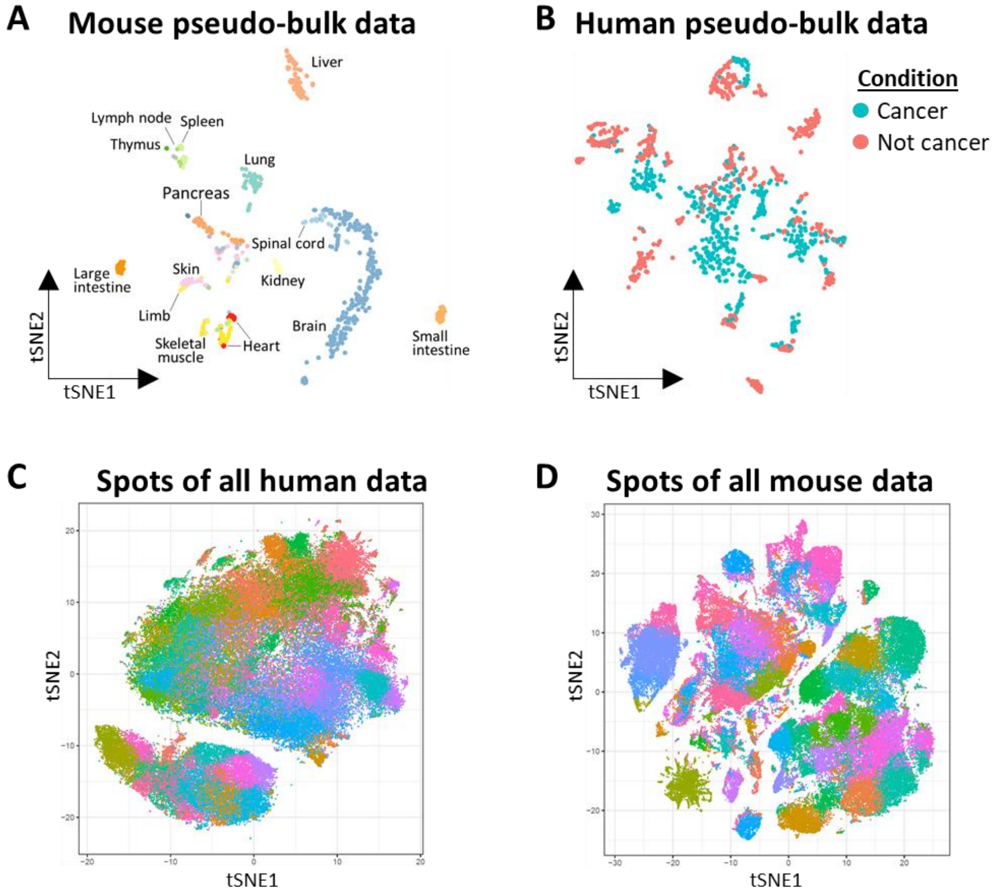

Suppl. Figure S2: Embeddings of Visium pseudobulk samples and Visium spots in DeepSpaceDB. **(A)** Embedding (tSNE plot) of mouse samples after processing to pseudobulk data. Colors indicate the tissue of origin. Prominent tissues are indicated. **(B)** Embedding (tSNE plot) of human samples after processing to pseudobulk data. Colors indicate whether the sample was obtained from a patient suffering from cancer (blue) or not (red). **(C-D)** Embedding (tSNE plot) of human **(C)** and mouse **(D)** spots. Colors indicate the 50 clusters obtained using k-means clustering. These are the clusters shown in Supplementary Figures S3,4 to which we assigned annotations.

(next page) **Suppl. Figure S3:** Properties of human spot clusters. **(A,B)** Overlap between spot clusters and tissue annotations (A), and between spot clusters and conditions annotations (B). Overlap between sets of spots was estimated using the Jaccard index, which was converted to Z scores through randomizations (see Methods). Red colors indicate strong overlaps. **(C)** Average scaled gene expression of selected differentially expressed genes of the 50 clusters. Annotations of clusters are indicated at the bottom of the figure.

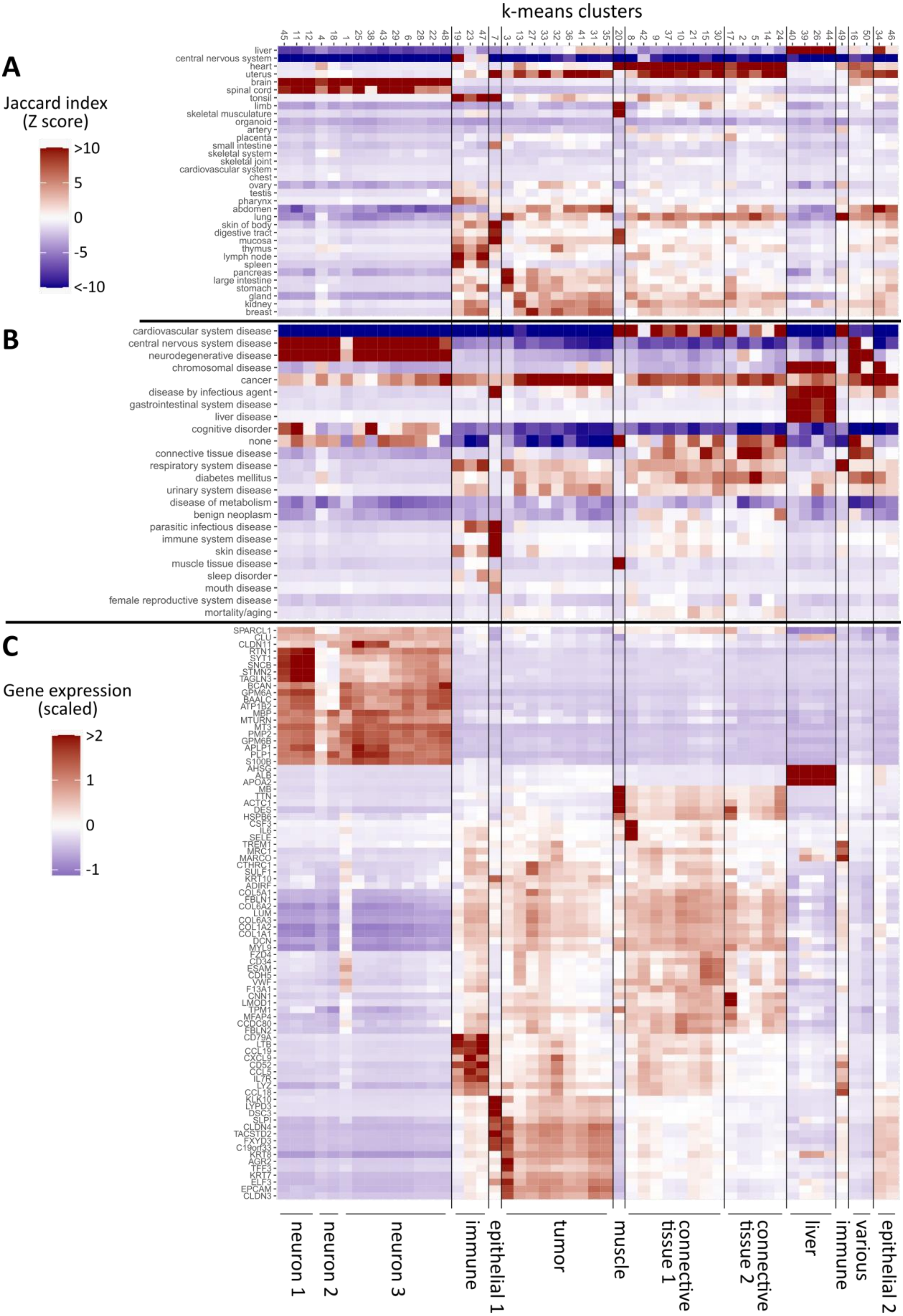

k-means clusters
45 11 12 4 18 1 25 38 43 29 6 28 22 48 19 23 47 7 3 13 27 33 32 36 41 31 35 20 8 42 9 37 10 21 15 30 17 2 5 14 24 40 39 26 44 49 16 50 34 46
A
Jaccard index (Z score)
>10
5
0
-5
<-10
liver
central nervous system
heart
uterus
brain
spinal cord
tonsil
limb
skeletal musculature
organoid
artery
placenta
small intestine
skeletal system
skeletal joint
cardiovascular system
chest
ovary
testis
pharynx
abdomen
lung
skin of body
digestive tract
mucosa
thymus
lymph node
spleen
pancreas
large intestine
stomach
gland
kidney
breast
B
cardiovascular system disease
central nervous system disease
neurodegenerative disease
chromosomal disease
cancer
disease by infectious agent
gastrointestinal system disease
liver disease
cognitive disorder
none
connective tissue disease
respiratory system disease
diabetes mellitus
urinary system disease
disease of metabolism
benign neoplasm
parasitic infectious disease
immune system disease
skin disease
muscle tissue disease
sleep disorder
mouth disease
female reproductive system disease
mortality/aging
C
Gene expression (scaled)
>2
1
0
-1
SPARCL1
CLU
CLDN11
RTN1
SYT1
SNCB
STMN2
TAGLN3
BCAN
GPM6A
BAALC
ATP1B2
MBP
MTURN
MT3
PMP2
GPM6B
APLP1
PLP1
S100B
AHSG
ALB
APOA2
MB
TTN
ACTC1
DES
HSPB6
CSF3
IL6
SELE
TREM1
MRC1
MARCO
CTHRC1
SULF1
KRT10
ADIRF
COL5A1
FBLN1
COL6A2
LUM
COL6A3
COL1A2
COL1A1
DCN
MYL9
FZD4
CD34
ESAM
CDH5
VWF
F13A1
CNN1
LMOD1
TPM1
MFAP4
CCDC80
FBLN2
CD79A
LTB
CCL19
CXCL9
CD52
CCL5
IL7R
LYZ
CCL18
KLK10
LYPD3
DSC3
SLPI
CLDN4
TACSTD2
FXYD3
C19orf33
KRT8
AGR2
TFF3
KRT7
ELF3
EPCAM
CLDN3
neuron 1
neuron 2
neuron 3
immune
epithelial 1
tumor
muscle
connective tissue 1
connective tissue 2
liver
immune
various
epithelial 2

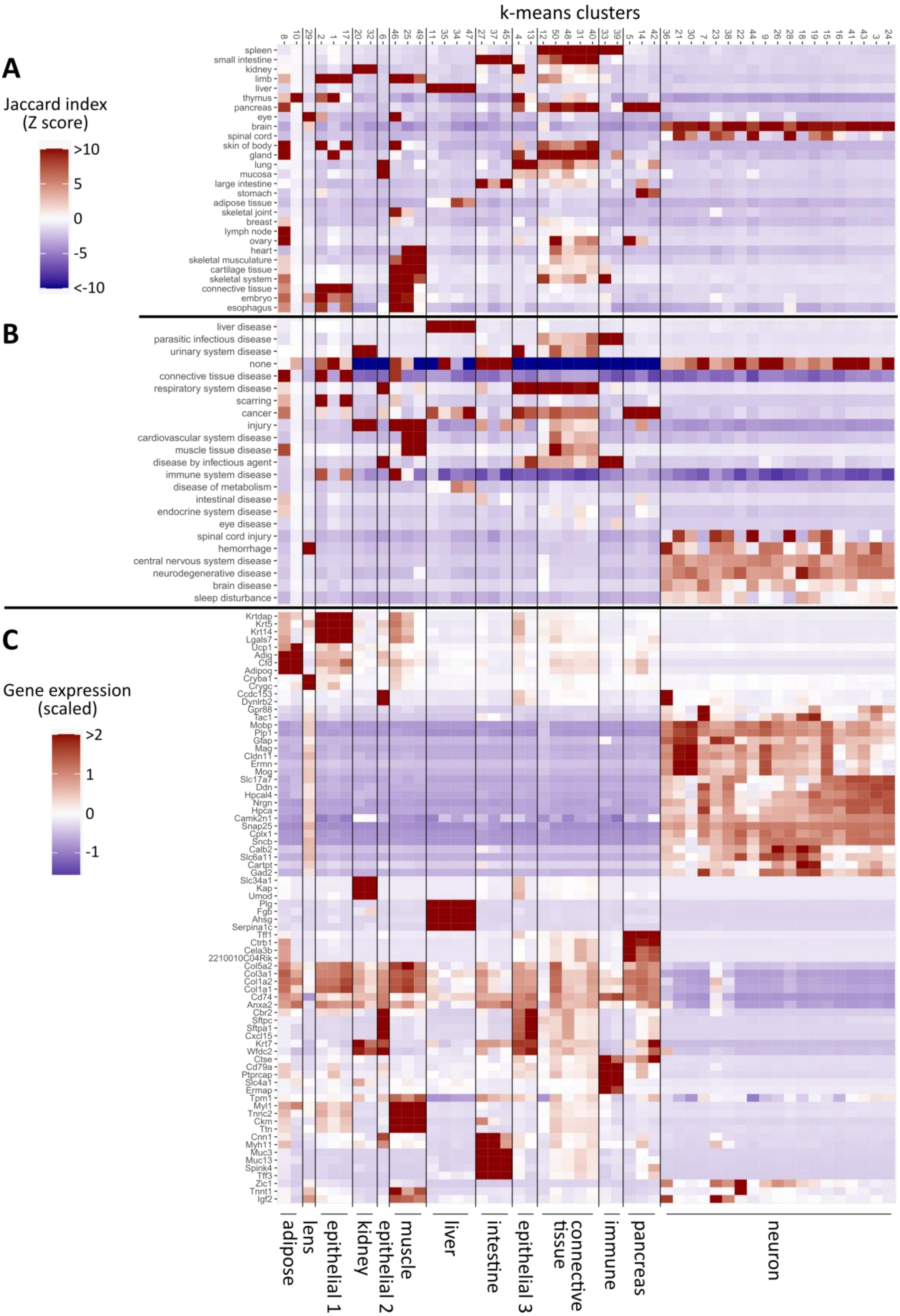
k-means clusters
A
Jaccard index (Z score)
>10
5
0
-5
<-10
spleen
small intestine
kidney
limb
liver
thymus
pancreas
eye
brain
spinal cord
skin of body
gland
lung
mucosa
large intestine
stomach
adipose tissue
skeletal joint
breast
lymph node
ovary
heart
skeletal musculature
cartilage tissue
skeletal system
connective tissue
embryo
esophagus
B
liver disease
parasitic infectious disease
urinary system disease
none
connective tissue disease
respiratory system disease
scarring
cancer
injury
cardiovascular system disease
muscle tissue disease
disease by infectious agent
immune system disease
disease of metabolism
intestinal disease
endocrine system disease
eye disease
spinal cord injury
hemorrhage
central nervous system disease
neurodegenerative disease
brain disease
sleep disturbance
C
Gene expression (scaled)
>2
1
0
-1
adipose
lens
epithelial 1
kidney
epithelial 2
muscle
liver
intestine
epithelial 3
connective tissue
immune
pancreas
neuron

(previous page) **Suppl. Figure S4:** Properties of mouse spot clusters. **(A,B)** Overlap between spot clusters and tissue annotations (A), and between spot clusters and conditions annotations (B). Overlap between sets of spots was estimated using the Jaccard index, which was converted to Z scores through randomizations (see Methods). Red colors indicate strong overlaps. **(C)** Average scaled gene expression of selected differentially expressed genes of the 50 clusters. Annotations of clusters are indicated at the bottom of the figure.

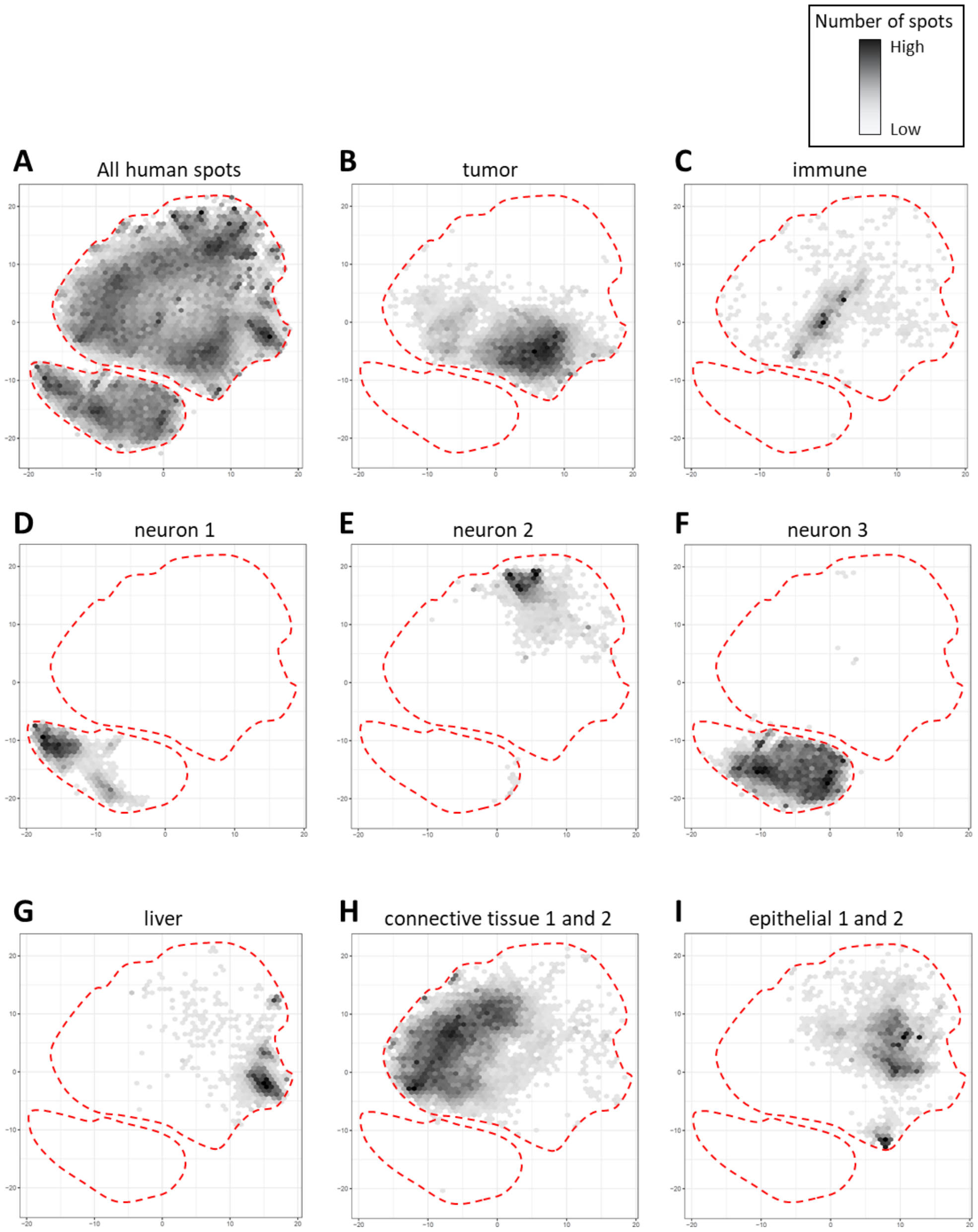

Suppl. Figure S5: Examples of the distribution of annotated human spot clusters. Each plot shows the distribution of spots within a 2D embedding (tSNE plot). This is the same embedding as shown in Suppl. Fig. S2C. To better reflect the density of spots, the space was divided into hexagonal bins, and the number of spots per bin is indicated by the intensity of the color (black: high, white: low). The red dotted lines show the rough contours of all spots in the 2D space as shown in panel (A), to facilitate comparison of distributions between panels. **(A)** Distribution of all human spots. **(B-I)** A number of example annotations and the distribution of spots with these annotations. The annotation is shown on top of each plot.

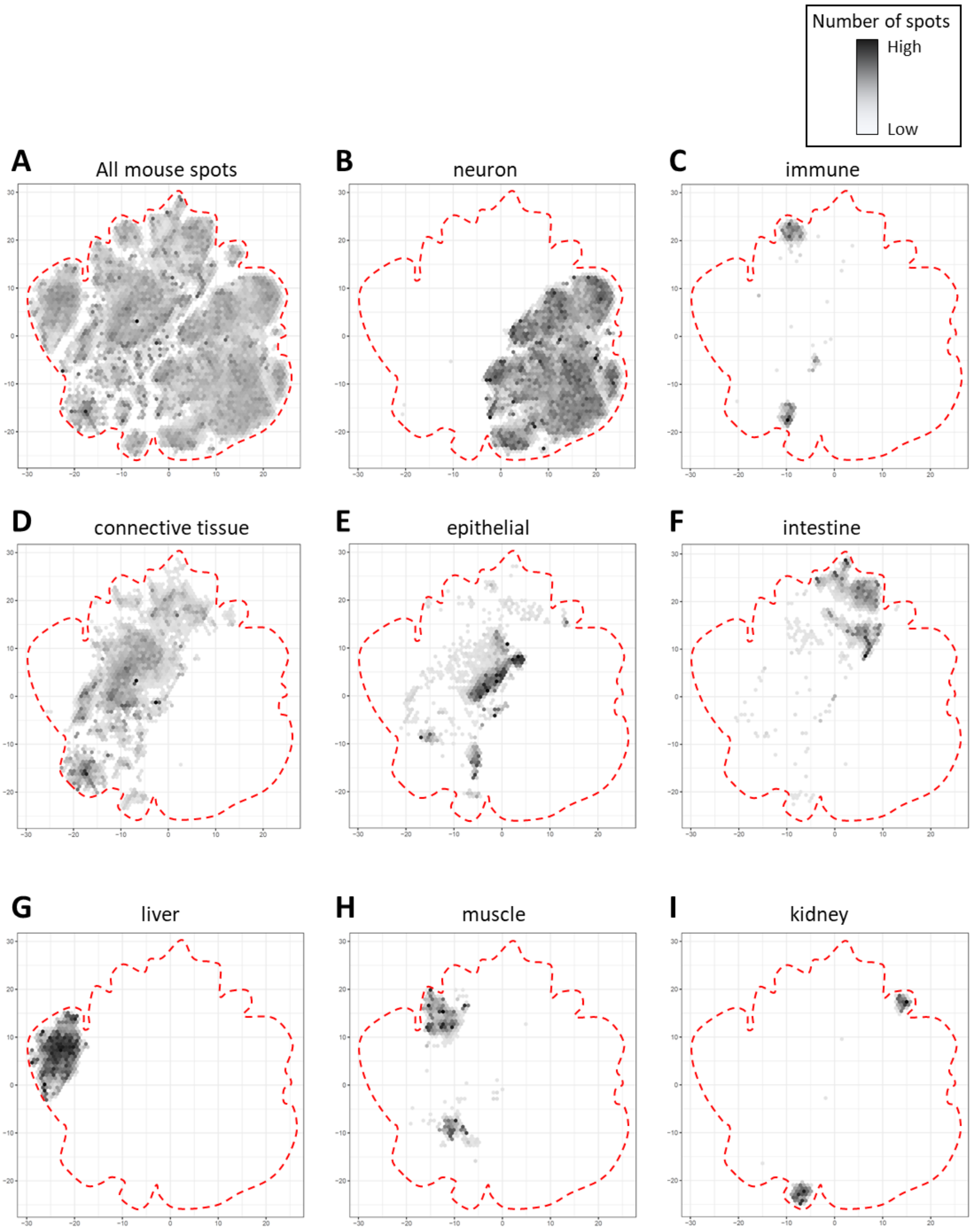

Suppl. Figure S6: Examples of the distribution of annotated mouse spot clusters. Each plot shows the distribution of spots within a 2D embedding (tSNE plot). This is the same embedding as shown in Suppl. Fig. S2D. To better reflect the density of spots, the space was divided into hexagonal bins, and the number of spots per bin is indicated by the intensity of the color (black: high, white: low). The red dotted lines show the rough contours of all spots in the 2D space as shown in panel (A), to facilitate comparison of distributions between panels. **(A)** Distribution of all mouse spots. **(B-I)** A number of example annotations and the distribution of spots with these annotations. The annotation is shown on top of each plot.

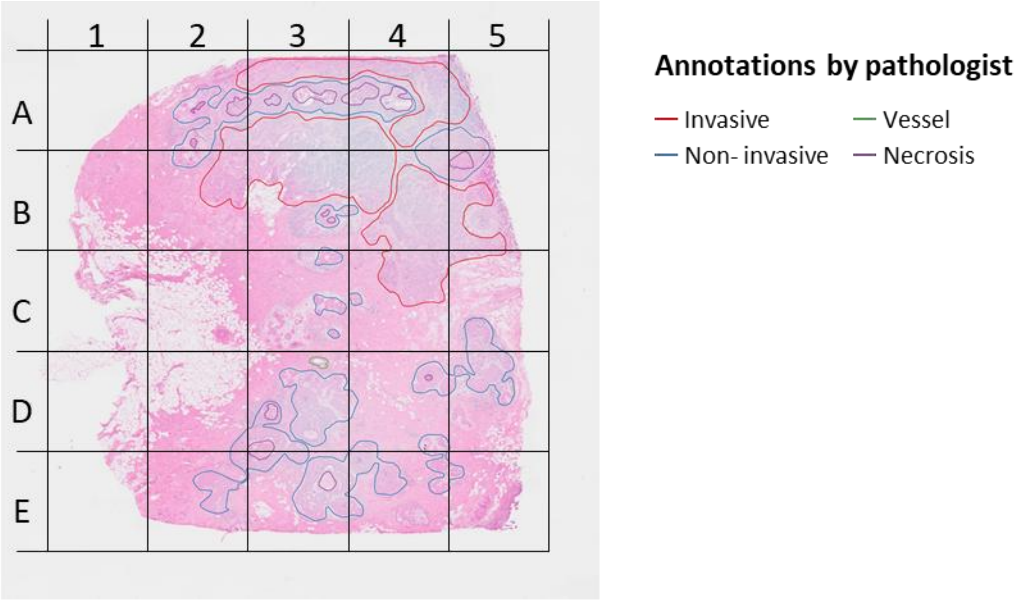

Row	Column	Annotations by LLM
A	1	Empty
A	2	Possible hyperplasia and irregular glandular structures.
A	3	The image shows malignant cell clusters with surrounding stromal invasion.
A	4	Possible abnormal glandular structures.
A	5	Not enough tissue to assess pathology.
B	1	Not enough tissue to identify specific pathological features.
B	2	Notable fibrosis and adipose tissue presence.
B	3	Indicators of invasive carcinoma with abnormal glandular structures.
B	4	Dense stroma, less glandular structure, possible tumor infiltration.
B	5	Empty
C	1	Empty
C	2	Presence of adipose tissue and a dense cluster of dark-staining cells indicating possible tumor.
C	3	Signs of invasive ductal carcinoma.
C	4	Signs of pathology: irregular cell clusters, increased stromal density, and presence of adipose tissue.
C	5	Not enough tissue to describe features or pathology.
D	1	Empty
D	2	Adipose tissue along with fibrosis. Insufficient details to comment on malignancy.
D	3	Signs of ductal carcinoma; irregular gland formation, cellular atypia.
D	4	Adipose tissue and possible glandular structures. No obvious pathology signs observable.
D	5	Presence of irregular glandular structures and desmoplastic stroma.
E	1	Empty
E	2	Visible glandular structures, possible signs of invasive carcinoma.
E	3	Lobular carcinoma indicated by irregular lobule structures.
E	4	Malignant cells, dense pink stroma, glandular structures, possible desmoplastic reaction.
E	5	No significant tissue features or pathology observed.

Suppl. Figure S7: LLM-based image annotations of the breast cancer sample. Top: The H&E image of the current sample with annotations by a human expert. The 5-by-5 grid used for the LLM-based image annotation is superimposed on the H&E image. Bottom: A table showing the LLM-based annotation for each element in the 5-by-5 grid.

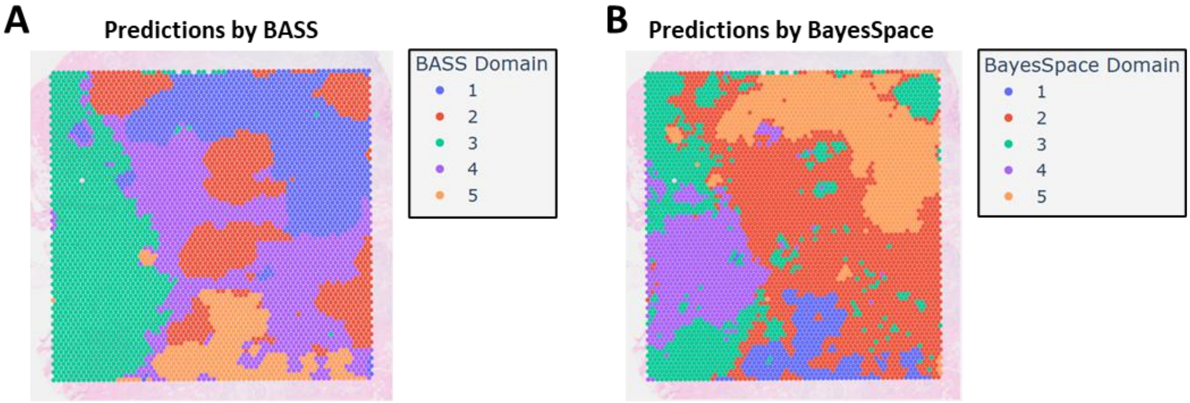

Suppl. Figure S8: Spatial domains predicted by BASS **(A)** and BayesSpace **(B)**. In both panels, colors reflect five predicted spatial domains. Predictions of both methods roughly reflect the presence of the tumor tissue (see Fig. 2E). On the DeepSpaceDB website, predictions for ten spatial domains are also available.

(next page) **Suppl. Figure S9:** Additional comparisons of gene expression between different regions within a sample. **(A)** Scatterplot of the average gene expression in set 1 (X axis) and set 3 (Y axis). A number of genes with large differences is indicated. **(B)** The spatial expression patterns of two selected genes are shown. *SLC12A2* has a higher expression in set 1 than in set 3. *FN1* has a higher expression in set 3 than in set 1. **(C)** Scatterplot of the average gene expression in set 2 (X axis) and set 3 (Y axis). A number of genes with large differences is indicated. **(D)** The spatial expression patterns of two selected genes are shown. *FASN* has a higher expression in set 2 than in set 3. *IGHA1* has a higher expression in set 3 than in set 2. **(E-G)** Comparison of biological process activities between two clusters. **(E)** The spot clustering result of this sample. Clusters 2 and 9 roughly cover the invasive and non-invasive tumor tissue in the sample, respectively (see Fig. 2E). **(F)** A volcano plot visualization of the comparison of pathway processes. The X axis shows the difference in mean pathway actitivities in the two clusters, and the Y axis the adjusted p-values (-$\log_{10}$ values) of a t-test comparing the activities in the two clusters. Three processes with relatively large differences of activity are indicated, and their activity patterns are visualized in **(G)**.

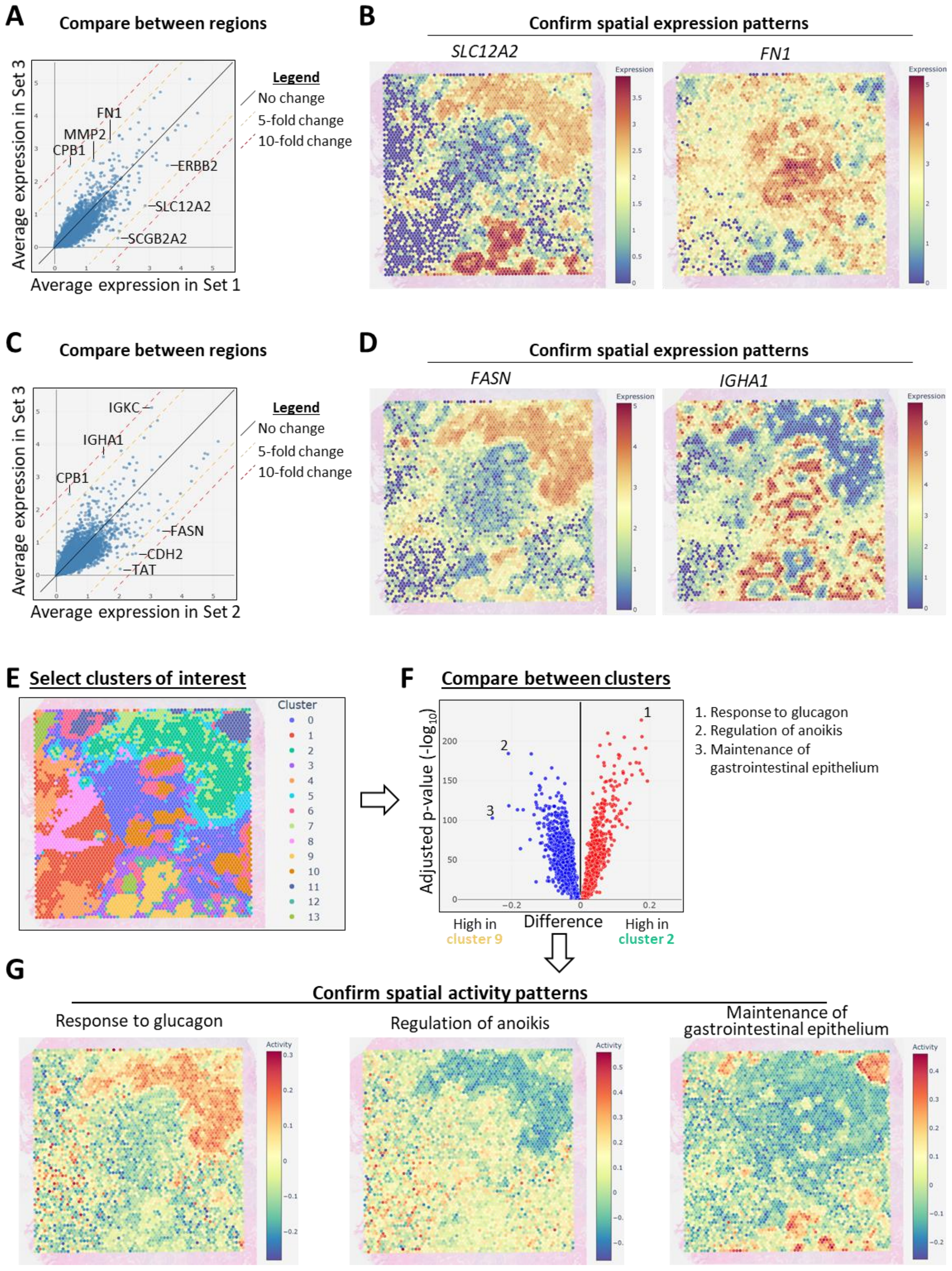
A
Compare between regions
Average expression in Set 3
Average expression in Set 1
FN1
MMP2
CPB1
ERBB2
SLC12A2
SCGB2A2
Legend
No change
5-fold change
10-fold change
B
Confirm spatial expression patterns
SLC12A2
FN1
Expression
C
Compare between regions
Average expression in Set 3
Average expression in Set 2
IGKC
IGHA1
CPB1
FASN
CDH2
TAT
Legend
No change
5-fold change
10-fold change
D
Confirm spatial expression patterns
FASN
IGHA1
Expression
E
Select clusters of interest
Cluster
F
Compare between clusters
Adjusted p-value (-log10)
Difference
High in cluster 9
High in cluster 2
1. Response to glucagon
2. Regulation of anoikis
3. Maintenance of gastrointestinal epithelium
G
Confirm spatial activity patterns
Response to glucagon
Regulation of anoikis
Maintenance of gastrointestinal epithelium
Activity

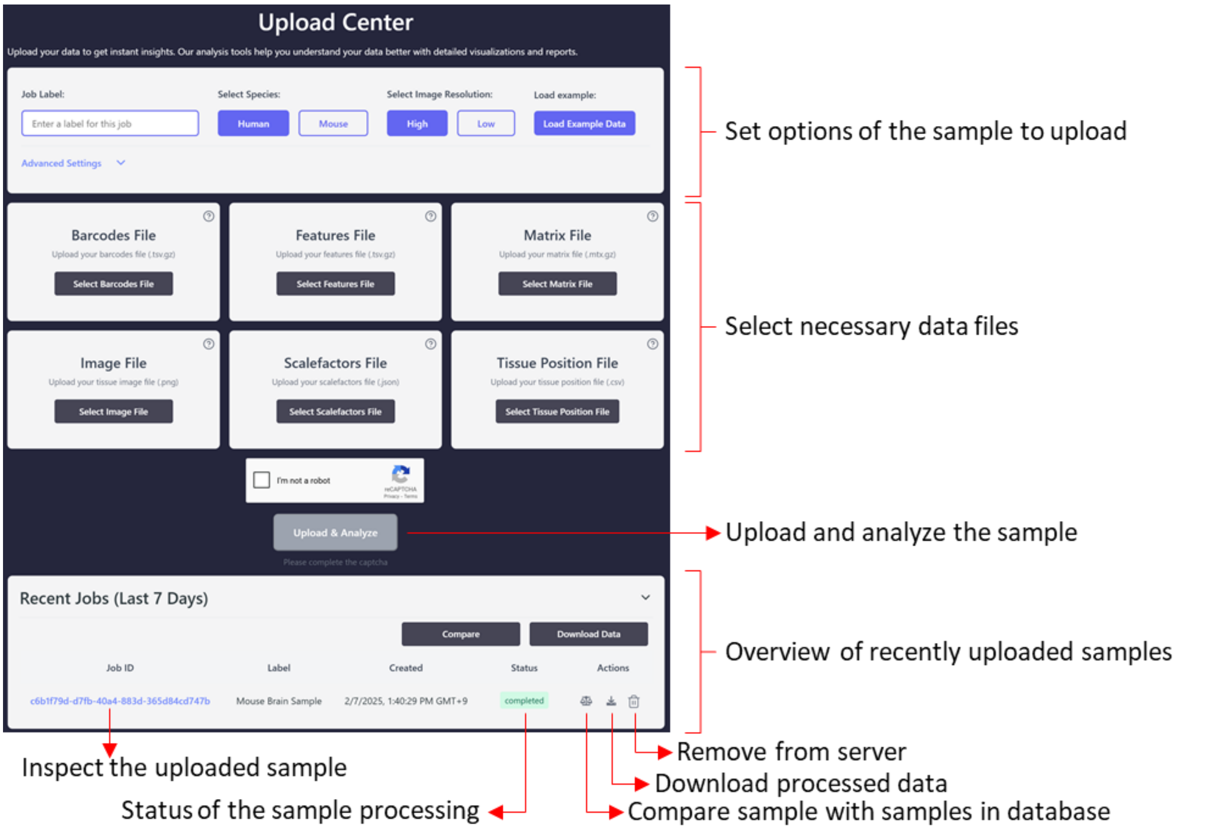

Suppl. Figure S10: Summary of the Upload Center of DeepSpaceDB. Users can upload Visium samples and process them in a similar way to the samples included in the DeepSpaceDB database. Users can give a job label, select the species and the image resolution of the uploaded image data. A human or mouse example sample can also be used. The data to upload consists of output files of the 10X Genomics Space Ranger software: 1) a barcode file, 2) a feature file, 3) a matrix file, 4) an image file, 5) a scalefactors file, and 6) a tissue position file. After uploading these data files, their content is checked on our server, and – if no problems are found – the data is submitted by our job scheduling software for processing. The status of the sample will be listed under "Recent jobs". After the processing has been completed, the uploaded sample can be inpected on the server. Alternatively, the sample can be compared with other samples, the processed data can be downloaded, or the sample can be deleted. Uploaded samples are automatically deleted after some time. Each uploaded sample is accessible through a unique URL, which can be shared with collaborators. Data is not visible or accessible to others without the unique URL. Uploaded data is not included in the DeepSpaceDB database, and will not be collected or retained on our side.
